# Editorial: Women in science: pharmacological treatment of pain

**DOI:** 10.3389/fpain.2023.1231281

**Published:** 2023-06-27

**Authors:** Erika Ivanna Araya, Juliana Geremias Chichorro

**Affiliations:** ^1^ Department of Medicine, Headache and Neurological Pain Research Group, Vall d’Hebron Research Institute, Universitat Autònoma de Barcelona, Barcelona, Spain; ^2^ Department of Pharmacology, Biological Sciences Sector, Federal University of Parana, Curitiba, Brazil

**Keywords:** chronic pain, depression, ketamine, lidocaine, CR4056, LPAR5 antagonist

**Editorial on the Research Topic** Women in science: pharmacological treatment of pain

Chronic pain is a common and distressing problem that affects about 30% of the population worldwide (1). The International Association for the Study of Pain classified chronic pain according to the International Classification of Diseases (ICD-11) of the World Health Organization (2) in chronic primary pain, such as fibromyalgia, complex regional pain syndrome, chronic migraine, and non-specific low-back pain; and chronic secondary pain, which is pain linked to an underlying condition. Additionally, chronic pain is classified according to severity, temporal progression, and evidence of psychological and social factors. Proper management of chronic pain is a challenge and usually requires a multidisciplinary approach, since it is often related to poor quality of life and the development of comorbidities, such as anxiety, depression and insomnia (3). Therefore, this research topic addresses several strategies in clinical and preclinical settings carried out by women scientists, first and/or last authors, to cover new potential therapies to relieve chronic pain.

Chronic pain treatment includes various pharmacological groups, such as anticonvulsants, antidepressants, nonsteroidal anti-inflammatory drugs, opioids, alone or in combination, among others. However, in most cases, patients suffering from chronic pain are refractory to multiple treatments and opioid abuse is often observed, leading to serious complications (4). As a result, different strategies have been studied to reduce opioid consumption in patients with chronic pain. On this research topic, a study published by Zavala et al. compared opioid consumption in patients with vaso-occlusive crises of sickle cell disease (SCD) undergoing additional lidocaine or ketamine infusion during painful crises with those using morphine monotherapy. Lidocaine is a sodium channel blocker that modulates the excitability of neurons peripherally, but also acts at the spinal cord and upper spinal cord level after systemic injection. There is evidence that lidocaine may modulate G protein-coupled receptors and ligand-gated channel receptors, which among other mechanisms, participate in its analgesic effect (5). As such, lidocaine infusion has emerged as a stabilizing agent for opioid doses in patients during painful crises. On the other hand, ketamine is a dissociative anesthetic widely used for induction and maintenance of anesthesia, which has been used in subanesthetic doses for the treatment of chronic pain and depression (6). This drug presents several mechanisms of action, notwithstanding, the main analgesic effect is related to N-methyl-D-aspartate (NMDA) receptor antagonism (7). The authors emphasize that SCD patients often reach adulthood as opioid-tolerant due to prolonged use of opioids to treat chronic pain and acute pain exacerbations. This study showed that both ketamine and lidocaine infusion was able to reduce mean daily morphine consumption by 28% and 23%, respectively. This finding provided a useful therapeutic approach to improve analgesia and decrease opioid consumption, which may be extremely relevant, especially for patients in need of long-term opioid use. It is now widely accepted that depressive comorbid symptoms are associated with chronic pain conditions (8) and poor treatment outcomes (9). It has been shown that the pharmacological properties of ketamine, for instance, modulation of glutamatergic activity, activation of the opioid system and regulation of the inflammatory response, cause anti-depressant effects, leading to relief of emotional pain, in addition to physical pain (10, 11). In this regard, another study presented in this research topic Batievsky et al. provided a first insight into the effect of ketamine use by two different therapeutic approaches in the management of chronic pain and comorbid major depressive disorder (MDD). In this report, the severity of pain, depression, and anxiety were reduced by ketamine in all patients under both approaches. However, the psychedelic approach (i.e., intramuscular high dose) was more likely to alleviate chronic pain and comorbid MDD than the psycholytic approach (i.e., sublingual low dose). This finding may serve as a basis for further studies with the aim of optimizing the use of ketamine in chronic pain patients.

NMDA receptors have been long considered a target for neuropathic pain, but high-affinity antagonists, such as ketamine, display severe and frequent side effects that limit their clinical use (12, 13). Thus, studies aiming to identify NMDA-interacting drugs that attenuate abnormal NMDA receptors activity with minimal impairment of their physiological function may lead to novel analgesics with better safety profile. In this regard, the study by Puja et al. showed that CR4056, an imidazoline-2 receptor ligand, modulate NMDA receptors activity both, in cell culture and spinal cord slices. The authors performed a series of experiments that indicate that CR4056 is a reversible, non-competitive antagonist of NMDA receptors, with higher potency and efficacy on receptors containing NR2B subunit. Although drugs that preferentially target this subunit are considered safer, the contribution of NR2B receptors to chronic pain is still a matter of debate, due to limited evidence of effects of NR2B antagonists at the spinal cord level (12, 14). Several studies have reported potent analgesic effect *in vivo* of CR4056 in different models of pain, in addition to evidence of analgesic activity in humans (15–17). Thus, mechanistic studiesare clearly warranted for a better understanding of CR4056 analgesic effect and its potential clinical indications.

Chronic pain has different etiologies and each form may involve distinct components and key cellular players. In neuropathic and inflammatory pain conditions, some studies identified that high levels of Lysophosphatidic acid (LPA) was linked to pathological progression of the disease, an effect that was later demonstrated to be mediated mainly by LPAR1 and LPAR5 receptors [for review see (18)]. One last study included in this research topic Langedijk et al. showed that a compound developed as a LPAR5 antagonist (Cpd3) showed antinociceptive effect *in vivo* in models of inflammatory pain, corroborating previous observations in inflammatory and neuropathic pain models (19, 20). However, unexpectedly, Cpd3 induced significant scratch activity in mice, an effect showed to be independent of TRPA1 and TRPV1 receptors. These finding contrast previous observations that LPA-induced itch was mediated by LPAR5/TRPV1/TRPA1 signaling pathways (21). These discrepant results need further investigation, but may be related to lack of selectivity of Cpd3 for LPAR5 or the complex interactions between itch and pain signaling pathways.

Collectively, these studies demonstrate the complexity of chronic pain management and highlight the efforts and challenges faced in the search for novel therapeutic targets.
